# A Narrative Review on GLP-1 Receptor Agonists for Obesity in Older Women: Maximizing Weight Loss While Preserving Lean Mass

**DOI:** 10.3390/nu18040632

**Published:** 2026-02-14

**Authors:** Federica Moscucci, Francesco Baratta, Daniele Pastori, Danilo Menichelli, Anna Vittoria Mattioli, Sabina Gallina, Ilaria Lospinuso, Susanna Sciomer, Gianfranco Piccirillo, Giovambattista Desideri

**Affiliations:** 1 Geriatric Unit, DAI of Internal Medicine and Medical Specialties, Azienda Ospedaliera Universitaria Policlinico Umberto I, Viale del Policlinico n.155, 00185 Rome, Italy; lospinuso.i@gmail.com (I.L.); gianfranco.piccirillo@uniroma1.it (G.P.); giovambattista.desideri@uniroma1.it (G.D.); 2 Department of Clinical Medicine, Life, Health and Environmental Sciences, University of L’Aquila, 67100 L’Aquila, Italy; 3 Department of Medical and Cardiovascular Sciences, University of Rome “Sapienza”, 00161 Rome, Italy; daniele.pastori@uniroma1.it; 4 Istituto di Ricovero e Cura a Carattere Scientifico (IRCCS) Neuromed, 86077 Pozzilli, Italy; 5 Department of General Surgery, Surgical Specialty and Anesthesiology Paride Stefanini, Sapienza University of Rome, 00161 Rome, Italy; danilo.menichelli@uniroma1.it; 6 Department of Quality of Life, University of Bologna-Alma Mater Studiorum, 40126 Bologna, Italy; annavittoria.mattioli@unibo.it; 7 Department of Neuroscience, Imaging and Clinical Sciences, “G. d’Annunzio” University of Chieti-Pescara, 66100 Chieti, Italy; sabina.gallina@unich.it; 8 University of Rome “Sapienza”, 00185 Rome, Italy; susanna.sciomer@uniroma1.it

**Keywords:** obesity, old women, cardiovascular prevention, GLP-1 receptor agonist, sarcopenia prevention, lifestyle for prevention

## Abstract

Obesity in women over 65 represents a growing clinical challenge, particularly due to its association with increased risks of cardiovascular disease, osteoarthritis, frailty, sleep-breathing disorders, and sarcopenia. The prevalence of obesity in this demographic is compounded by age-related metabolic changes and declining physical activity. In this context, glucagon-like peptide-1 receptor agonists (GLP-1 RAs), such as semaglutide and tirzepatide, have emerged as promising pharmacological treatments for weight loss, showing substantial efficacy in reducing body weight and improving metabolic health. However, their use in older populations warrants careful consideration due to the potential risk of muscle loss, which may exacerbate sarcopenia and frailty. This review synthesizes current evidence on the efficacy of GLP-1 RAs for weight loss in older women, exploring both the metabolic benefits and potential risks, particularly with regard to muscle mass preservation. We discuss the mechanisms behind muscle loss associated with GLP-1 RAs, focusing on the balance between fat reduction and the preservation of lean body mass. In phase 3 trials, women aged ≥ 65 years achieved sustained weight loss of 10–20%, with consistent cardiometabolic improvements. Furthermore, we propose practical strategies to mitigate sarcopenia, including physical activity interventions, dietary modifications, and combination therapies aimed at maintaining muscle strength while promoting weight loss. By examining clinical trial data, real-world evidence, and physiological mechanisms, this narrative review aims to provide a comprehensive framework for personalized and safe therapeutic decision-making, addressing the unique needs of older women with obesity.

## 1. Introduction

The global prevalence of obesity in older adults is rising at an alarming rate, with profound implications for both physical and psychological well-being. Obesity is now recognized as a major contributor to the increasing burden of chronic diseases, such as cardiovascular disease, type 2 diabetes, osteoarthritis, and several malignancies [1,2]. For older adults, these conditions can significantly impair their quality of life, independence, and mobility [3]. In postmenopausal women, the risk of obesity-related health complications is further heightened by the hormonal changes associated with menopause [4]. These changes, particularly the decline in estrogen levels, have a marked effect on fat distribution and metabolism, leading to an increased propensity for abdominal fat accumulation around the abdomen [5] in up to one-third of women aged ≥ 65 years. This shift in fat distribution is linked to a higher risk of developing metabolic syndrome, cardiovascular disease, and insulin resistance, further complicating obesity management [4,5].

In addition to hormonal changes, aging is accompanied by a gradual loss of muscle mass and strength, a condition known as sarcopenia. This decline in muscle mass, combined with the accumulation of visceral fat, creates a dangerous interplay that exacerbates frailty and reduces functional capacity [6,7]. As muscle loss increases, older adults experience a decrease in resting metabolic rate, making weight management more challenging. This phenomenon, along with a reduction in physical activity levels due to chronic pain, fatigue, or physical limitations, leads to a vicious cycle of weight gain, decreased mobility, and further muscle loss.

Sarcopenic obesity in older women arises from a complex interplay of molecular mechanisms that affect both adipose and muscle tissues. As detailed by Millán-Domingo et al. [8], these mechanisms include mitochondrial dysfunction, oxidative stress, and inflammatory pathways that contribute to muscle atrophy and fat accumulation.

Mitochondrial dysfunction leads to decreased energy production in muscle cells, impairing their ability to maintain mass and function [7,8]. This dysfunction is often accompanied by increased oxidative stress, which damages cellular components and exacerbates muscle degradation. Additionally, chronic low-grade inflammation, characterized by elevated levels of pro-inflammatory cytokines, disrupts muscle protein synthesis and promotes fat deposition [9].

The accumulation of lipids within muscle cells, a condition known as myosteatosis, further impairs muscle function and contributes to insulin resistance. This resistance hampers the anabolic response to nutrients, leading to a cycle of muscle loss and fat gain [10]. Moreover, hormonal changes associated with aging, such as decreased levels of estrogen and growth factors, exacerbate these processes by reducing muscle regeneration and promoting fat storage [9].

Understanding these molecular pathways is crucial for developing targeted interventions to prevent and manage sarcopenic obesity in older women [11]. Strategies that address mitochondrial health, reduce oxidative stress, and modulate inflammatory responses hold promise for mitigating the adverse effects of this condition [12].

While traditional lifestyle interventions such as diet modification and exercise remain the cornerstone of obesity treatment, their effectiveness in older populations is often limited. Many older adults face barriers to adhering to exercise regimens due to physical limitations, comorbidities, and other social determinants of health [13]. Furthermore, caloric restriction, which is commonly recommended for weight loss, may lead to negative consequences in older individuals, such as further muscle depletion, malnutrition, and a decline in functional capacity [14]. These challenges have led to a growing interest in pharmacologic interventions that can support weight loss while also mitigating the adverse effects of obesity in the aging population.

Among the pharmacologic options available, glucagon-like peptide-1 receptor agonists (GLP-1 RAs) have emerged as promising agents for obesity management. GLP-1 RAs, including semaglutide and tirzepatide, have demonstrated significant efficacy in promoting weight loss and improving metabolic health in younger populations [15]. These medications work by mimicking the action of GLP-1, a hormone that plays a central role in regulating appetite, insulin secretion, and glucose metabolism. By stimulating GLP-1 receptors in the brain, GLP-1 RAs help to reduce hunger and promote satiety, leading to reduced caloric intake and, subsequently, weight loss. Additionally, these agents enhance insulin sensitivity and have beneficial effects on blood sugar levels, further contributing to their appeal in the treatment of obesity and type 2 diabetes [16].

The use of GLP-1 RAs in geriatric populations, however, necessitates a more nuanced approach. While the evidence supporting the efficacy of GLP-1 RAs in younger adults is robust, their application in older individuals requires careful consideration of age-specific physiological, functional, and social factors [17]. Older women, in particular, may experience a higher risk of adverse effects such as muscle loss, dehydration, and gastrointestinal distress. Given the age-related changes in body composition and organ function, these individuals may also have a different pharmacokinetic profile compared to younger populations, which could affect both the efficacy and safety of GLP-1 RAs [18].

Furthermore, the therapeutic goals for managing obesity in older women extend beyond weight loss alone. While reducing excess weight is crucial, it is equally important to consider the preservation of muscle mass, mobility, and overall functional independence. Weight loss in older women must be carefully managed to avoid the unintended consequence of sarcopenia, a condition that further exacerbates frailty and increases the risk of falls, disability, and premature mortality [19]. Therefore, therapeutic strategies should aim not only to reduce adiposity but also to preserve or even enhance muscle mass, strength, and physical function. This holistic approach requires individualized care, considering the patient’s overall health status, functional capacity, and personal goals.

Fall prevention is another critical aspect of managing obesity in older women. Obesity is a known risk factor for falls and fractures due to its impact on balance, joint health, and muscle strength. However, weight loss, if not carefully managed, can also pose a risk to balance and strength, particularly if the loss occurs without concurrent efforts to preserve muscle mass [20]. Thus, the management of obesity in this population must balance the goal of reducing body weight with strategies to prevent falls and maintain physical independence. This may involve integrating resistance training and physical rehabilitation programs alongside pharmacologic interventions to optimize outcomes [21].

In light of these considerations, the use of GLP-1 RAs in older women requires a comprehensive, individualized approach that addresses not only weight loss but also the preservation of muscle mass, functional capacity, and overall quality of life.

## 2. Methods

A comprehensive literature search was conducted using PubMed, Scopus, and Web of Science (First access to data bases in 10 July 2025, last in 30 December 2025); the literature search covered publications from January 2015 to March 2025, with the final search conducted on 15 March 2025, in order to capture the most recent decade of evidence on GLP-1 receptor agonists and body composition outcomes in older adults. Search terms included combinations of “GLP-1 receptor agonists”, “semaglutide”, “tirzepatide”, “obesity”, “older adults”, “older women”, “sarcopenia”, and “body composition”. Randomized controlled trials, narrative and systematic reviews, and real-world observational studies were considered based on their relevance to weight loss efficacy, body composition changes, and functional outcomes in older adults, with particular attention to sex- and age-specific analyses when available. After screening for relevance and eligibility, a total of 62 articles were included in the final qualitative synthesis and form the evidentiary basis of this narrative review. Given the heterogeneity of study designs and outcomes, evidence was synthesized narratively.

## 3. Physiology and Mechanism of Action of GLP-1 Receptor Agonists (GLP-1 RAs)

The GLP-1 is an incretin hormone primarily secreted by the L-cells of the distal small intestine in response to nutrient ingestion. Its secretion is triggered by the presence of food, particularly carbohydrates and fats, and plays a critical role in regulating glucose homeostasis and energy balance. GLP-1 exerts multiple effects that are pivotal in maintaining glucose control and reducing food intake, which are beneficial for managing obesity and metabolic disorders such as type 2 diabetes [22].

One of the primary actions of GLP-1 is to enhance glucose-dependent insulin secretion. When blood glucose levels rise after eating, GLP-1 stimulates pancreatic beta cells to secrete insulin, thus promoting glucose uptake by peripheral tissues [23]. Conversely, GLP-1 suppresses glucagon secretion from pancreatic alpha cells, preventing excessive glucose production from the liver. This reduction in hepatic glucose output is crucial in preventing postprandial hyperglycemia. Additionally, GLP-1 slows gastric emptying, leading to prolonged satiety and reduced food intake [24]. These effects collectively help control postprandial blood glucose levels and contribute to overall weight loss.

The appetite-regulating actions of GLP-1 are mediated through its action on central appetite control centers in the brain, particularly the hypothalamus. GLP-1 receptor activation in these centers promotes feelings of fullness (satiety), leading to reduced caloric intake. This central action is an essential component of the weight loss effects observed with GLP-1 RAs, which mimic GLP-1’s physiological effects [16]. The central and peripheral actions of GLP-1, therefore, contribute to its dual benefits in weight management and glycemic control, making GLP-1 RAs effective agents for treating obesity and type 2 diabetes, particularly in older adults who are more prone to these conditions due to age-related physiological changes [24].

Pharmacological GLP-1 RAs, such as semaglutide and liraglutide, replicate the actions of natural GLP-1 by binding to GLP-1 receptors, which are widely distributed across various tissues, including the pancreas, gastrointestinal tract, brain, and cardiovascular system [25]. This broad distribution of GLP-1 receptors is key to the comprehensive metabolic effects of GLP-1 RAs. In addition to their appetite-suppressing effects, GLP-1 RAs improve insulin sensitivity by enhancing insulin release in response to meals and increasing glucose uptake by tissues, particularly muscle and adipose tissue [26]. Furthermore, GLP-1 RAs modulate inflammation by reducing pro-inflammatory cytokine production, a feature that could be particularly beneficial in the context of obesity and type 2 diabetes, which are often characterized by low-grade chronic inflammation [27].

In the cardiovascular system, GLP-1 RAs have been shown to exert cardioprotective effects. These include improved endothelial function, reduced blood pressure, and potentially a reduction in the incidence of cardiovascular events, including stroke and myocardial infarction [28]. These cardiovascular benefits are of particular importance in older adults with obesity, who are at increased risk for cardiovascular disease. The ability of GLP-1 RAs to improve both metabolic and cardiovascular outcomes makes them an attractive therapeutic option for older patients who are dealing with the dual challenges of obesity and aging-related comorbidities [29].

Tirzepatide, a novel dual GLP-1 and gastric inhibitory peptide (GIP) receptor agonist, offers an enhancement over traditional GLP-1 RAs by combining the actions of both incretin hormones. GIP, another incretin hormone, works synergistically with GLP-1 to promote insulin secretion and regulate lipid metabolism [30]. GIP has a unique ability to enhance insulin release in response to glucose while also promoting fat breakdown, which complements GLP-1’s actions on glucose control and appetite regulation. The dual agonism of tirzepatide results in more pronounced weight loss, greater satiety, and potentially superior glycemic control compared to GLP-1-only therapies. This synergistic effect on both insulin secretion and energy balance is particularly beneficial for patients with obesity and type 2 diabetes, offering an improved approach to managing these conditions in older adults who may be more resistant to traditional therapies [31].

Studies have shown that tirzepatide leads to significant reductions in body weight and improvements in glycemic control in both younger and older populations. According to a systematic review by Wang et al. [17], GLP-1 RAs, including tirzepatide, demonstrate efficacy and safety in older adults, with significant improvements in HbA1c levels, weight loss, and cardiovascular risk factors. Notably, tirzepatide’s dual action on GIP and GLP-1 receptors enhances its ability to manage obesity and type 2 diabetes, providing a comprehensive solution for older individuals facing metabolic and cardiovascular challenges [17]. This dual receptor activity makes tirzepatide an especially promising treatment for older patients, where the complexities of aging-related physiology, including changes in insulin sensitivity and adiposity, may necessitate more potent interventions.

In addition to the direct metabolic effects, GLP-1 RAs may help to mitigate sarcopenic obesity, a condition often observed in older adults, by improving muscle mass retention through better glucose utilization and enhancing fat metabolism [32]. Sarcopenic obesity, characterized by the combination of excess adiposity and muscle loss, is a significant concern for older women. The use of GLP-1 RAs, by modulating energy balance, reducing visceral fat, and possibly preserving lean mass, offers a multifaceted approach to addressing this condition [33]. As highlighted by Moiz et al. [16], GLP-1 RAs exert their effects on both central and peripheral pathways to regulate appetite, energy expenditure, and insulin sensitivity, which together contribute to weight loss and improved muscle function, reducing the risk of frailty and maintaining functional independence in older women.

Overall, GLP-1 RAs, particularly tirzepatide, represent a promising therapeutic option for managing obesity and type 2 diabetes in older adults. The dual action of GLP-1 and GIP receptor agonism provides an enhanced effect on insulin sensitivity, energy regulation, and weight loss, making it an ideal treatment for addressing the complex metabolic challenges faced by the aging population [30,31,32,33]. These agents offer significant benefits beyond weight loss, improving glycemic control, reducing cardiovascular risk, and potentially alleviating the burdens of sarcopenic obesity, thus enhancing overall health and quality of life for older adults.

## 4. Efficacy of GLP-1 Receptor Agonists in Women Aged > 65 Years

### 4.1. Evidence from Randomized Controlled Trials

The efficacy of GLP-1 receptor agonists (GLP-1 RAs) in older adults has been robustly demonstrated in randomized controlled trials (RCTs) [34], providing strong evidence for their use in managing obesity and related comorbidities in individuals aged over 65. Two major trial programs, the STEP program for semaglutide [35,36,37,38,39] and the SURMOUNT trials [40,41,42,43] for tirzepatide, offer critical insights into the benefits of these therapies in older populations.

In the STEP program [35,36,37,38,39], which assessed the effects of semaglutide, two key studies—STEP 1 [35] and STEP 5 [39]—examined the weight loss effects in older adults. In both trials, subanalyses specifically focusing on women aged 65 years and older showed that these individuals experienced significant reductions in body weight, comparable to those seen in younger cohorts. On average, weight loss in this age group ranged from 10–15%, with a slight attenuation of this effect in participants aged over 70 years. This attenuation may be attributed to age-related metabolic changes, including reduced caloric expenditure, muscle mass, and hormonal shifts that influence weight regulation. Despite this attenuation, the improvements observed in metabolic parameters, such as HbA1c (glycated hemoglobin), blood pressure, and lipid profiles, were significant. Notably, STEP 5 [39] confirmed that these weight loss effects were durable, with sustained reductions in body weight and improvements in metabolic outcomes over a two-year period, which is particularly relevant for the management of chronic obesity in aging populations. The long-term efficacy observed in these trials reinforces the potential for GLP-1 RAs to manage obesity as a chronic condition in older women [Table 1]. In other words, weight reduction was observed during active treatment, while post-discontinuation follow-up indicated relative weight stability after treatment cessation, rather than further weight loss or rebound.

Similarly, the SURMOUNT-1 [40] and 2 [41] trials, which evaluated the effects of tirzepatide, revealed even greater weight loss efficacy, with mean reductions in body weight ranging from 15% to 21%. These trials also included older adults, showing that tirzepatide’s dual agonism (activating both GLP-1 and GIP receptors) may offer superior efficacy compared to single-agonist GLP-1 therapies. The sustained effects of tirzepatide were confirmed up to 72 weeks, highlighting its potential for long-term use in older adults managing obesity. In addition to weight loss, participants in these trials showed improvements in insulin sensitivity, greater reductions in fat mass, and modest improvements in physical functioning metrics, such as mobility and endurance. While the data on women over 75 years of age remains limited, the overall findings from these trials support the efficacy of tirzepatide in older women, particularly those with obesity and type 2 diabetes, a common comorbidity in this population [Table 1].

Across trials, approximately 25–40% of total weight loss has been attributed to lean mass reduction, underscoring the importance of muscle-preserving strategies in older women.

### 4.2. Real-World Evidence

Real-world evidence from observational studies has further corroborated the findings from randomized controlled trials, demonstrating that GLP-1 RAs are effective in achieving clinically meaningful weight loss in older women, particularly those with type 2 diabetes or cardiovascular comorbidities [44]. These studies highlight that older patients can achieve significant weight reduction, with many experiencing reductions in HbA1c, improved blood pressure, and better lipid profiles. In addition to metabolic improvements, real-world data indicate that older adults treated with GLP-1 RAs show reductions in healthcare utilization, such as fewer hospital admissions and a decrease in the need for medication adjustments, which ultimately enhances their health-related quality of life [45,46]. The ability of GLP-1 RAs to reduce the burden of chronic disease, improve glycemic control, and decrease healthcare costs presents a compelling case for their integration into clinical practice for older women, especially those with comorbid conditions like diabetes and hypertension [47].

However, the generalizability of trial data to multimorbid elderly patients remains a critical point of concern. While GLP-1 RAs have shown positive outcomes in controlled trial settings, real-world applications must consider the unique challenges that frail older adults face. For example, individuals with cognitive impairments, mobility limitations, or on polypharmacy regimens may experience difficulties with injectable therapies, which are a common form of administration for GLP-1 Ras [48]. The act of self-injecting, combined with the possible side effects of the medications, such as nausea and gastrointestinal discomfort, could pose significant barriers to adherence in these patients. Furthermore, appetite suppression, while beneficial for weight loss, may be less well-tolerated in frail older adults, particularly those at risk for malnutrition or those with low body mass.

In older women, the clinical issue is not “low body mass” as a contraindication, but rather reduced muscle reserves and/or low muscle function, particularly in the context of sarcopenic obesity, where excess adiposity coexists with impaired muscle strength and unfavorable body composition. This phenotype is especially relevant in women ≥65 years due to menopausal body-fat redistribution, anabolic resistance, and higher baseline risk of functional decline. To improve clinical applicability, a structured phenotyping and monitoring approach may be adopted using the ESPEN–EASO diagnostic algorithm, which prioritizes the assessment of muscle strength followed by confirmation of sarcopenic obesity through body-composition evaluation (e.g., DXA/BIA) with muscle mass normalized to body weight and concurrent evidence of excess adiposity, with subsequent staging according to functional and cardiometabolic complications [49].

As a result, clinicians must exercise caution when prescribing GLP-1 RAs to elderly patients, especially those with cognitive impairments or those who are frail, as the benefits must be carefully weighed against the potential risks [50].

Despite these challenges, the real-world evidence underscores the promise of GLP-1 RAs in managing obesity and its comorbidities in older women. With proper patient selection, monitoring, and adjustment of treatment plans, GLP-1 RAs can provide significant improvements in weight, glycemic control, and overall health outcomes. Future research should focus on identifying specific subgroups of older patients who are most likely to benefit from these therapies, as well as strategies to mitigate potential risks and optimize adherence in frail, multimorbid individuals. In sum, while GLP-1 RAs represent an important therapeutic option for managing obesity in older women, further evidence is needed to refine treatment approaches and ensure the safety and efficacy of these agents in the most vulnerable populations.

## 5. Muscle Loss: A Geriatric Concern

### 5.1. Sarcopenia and Aging

Aging is characterized by a progressive decline in skeletal muscle mass and function, a condition known as sarcopenia. This condition significantly increases the risk of falls, fractures, disability, and mortality [51]. Sarcopenia is considered a distinct geriatric syndrome and is particularly prevalent in older women, largely due to menopause-related hormonal changes, reduced physical activity levels, and the cumulative burden of chronic diseases [52]. The loss of muscle mass in this population is further exacerbated by anabolic resistance and low-grade chronic inflammation, often referred to as “inflammaging” [9]. These factors impair the muscle’s ability to respond effectively to anabolic stimuli, thus contributing to the progressive deterioration of muscle tissue.

Although sarcopenia has classically been regarded as a manifestation of undernutrition, in recent decades—and especially in both industrialized and developing countries—it has become increasingly linked to obesity, establishing a self-sustaining vicious cycle between these two interrelated conditions [19]. The effect of adiposity amplifies all the pathophysiological mechanism of the inflammaging sustaining the lean mass loss [9,19].

### 5.2. GLP-1 RAs and Lean Mass Loss

Clinical trials have demonstrated that approximately 25–40% of the total weight loss observed with GLP-1 receptor agonists (GLP-1 RAs) is attributable to the loss of lean mass, including muscle. This proportion of lean mass loss is of particular clinical concern in older women, particularly those with pre-existing sarcopenia or low baseline muscle reserves [53]. The use of tirzepatide, which induces greater total weight reduction, may be associated with even higher absolute losses in lean mass. Although some studies suggest that GLP-1 RAs preferentially reduce visceral fat, the risk of functional decline remains significant, as muscle loss may impair overall physical function [54].

To our best knowledge to the date, there is only one clinical study investigating the impact of GLP-1RAs on sarcopenic obesity. The SEMALEAN study, conducted in 106 patients, predominantly female, demonstrated a significant reduction in the prevalence of sarcopenic obesity, decreasing from 49% at baseline to 33% after 12 months of semaglutide treatment [55]. Moreover, the reduction in sarcopenic obesity prevalence occurred despite an initial decline in absolute lean mass during the early phase of semaglutide treatment (approximately −3 kg), which subsequently stabilized over follow-up, while indicators of muscle function showed improvement. This improvement was accompanied by favorable changes in body composition assessed by dual-energy X-ray absorptiometry (DEXA), including a significant reduction in total and visceral fat mass, while absolute lean mass loss was relatively limited and proportionally lower than fat mass reduction. Consequently, appendicular lean mass indexed to body weight and fat-free mass percentage showed stabilization or relative improvement over follow-up. In addition, preclinical data suggest that GLP-1 receptor agonists not only mitigate weight gain but also attenuate muscular atrophy and improve mitochondrial function in skeletal muscle, potentially disrupting the vicious cycle linking adiposity and sarcopenia [56]. These effects appear to be mediated, at least in part, by reductions in systemic and local low-grade inflammation and improvements in metabolic and mitochondrial efficiency [56,57,58].

### 5.3. Functional Implications

The loss of muscle mass has profound functional implications, translating into decreased strength, endurance, and overall physical capacity. Studies utilizing dual-energy X-ray absorptiometry (DEXA) and physical performance tests—such as the Short Physical Performance Battery (SPPB) and handgrip strength assessments—have documented declines in functional status in a subset of patients receiving GLP-1 RAs, particularly in those who do not engage in concurrent physical activity interventions [57]. A decline in physical function undermines the benefits of weight loss and may lead to increased dependency, institutionalization, and elevated mortality risk. Therefore, it is crucial to consider the potential adverse effects of muscle loss when prescribing GLP-1 RAs to older adults, particularly in those with pre-existing sarcopenia or who are at risk of functional decline [33].

## 6. Strategies to Mitigate Muscle Loss

### 6.1. Sarcopenia Case Finding, Comprehensive Geriatric Assessment and Monitoring

A comprehensive approach to managing muscle loss in older women requires regular and systematic monitoring of nutritional status, muscle strength, and physical performance. Routine assessments, such as handgrip strength and gait speed, are essential indicators of muscle function and overall physical health. These tests provide valuable insights into an individual’s ability to perform daily activities and their risk for functional decline, frailty, and falls. Incorporating these assessments into clinical practice ensures that interventions are tailored to the individual’s specific needs, which is especially important for the aging population, where baseline health conditions can vary significantly [58].

According to the European Working Group on Sarcopenia in Older People 2 (EWGSOP2), the identification of potential cases should begin when an individual shows symptoms or signs suggestive of sarcopenia—such as frequent falls, general weakness, slowed walking speed, difficulty standing up from a chair, or unintentional weight and muscle loss. In these situations, EWGSOP2 advises using the SARC-F questionnaire to gather patients’ self-reported information on typical sarcopenia-related issues [59]. If the SARC-F score suggests “probable sarcopenia,” EWGSOP2 recommends proceeding with a stepwise diagnostic pathway. This involves assessing muscle strength, confirming the condition through imaging techniques, and determining its severity. This structured approach follows the FACS model: Find cases, Assess, Confirm, and determine Severity [59].

However, certain studies have shown that SARC-F performs less reliably in individuals with sarcopenic obesity [60]. As a result, some authors recommend using adapted screening approaches that incorporate additional measures such as mid-arm circumference (MAC) and/or calf circumference (CC) to improve detection accuracy.

In our view, it could be reasonable to perform preliminary screening before initiating GLP-1 receptor agonist therapy for obesity in older women. Using modified versions of the SARC-F or directly assessing muscle strength—such as through handgrip testing—may be appropriate. This approach is justified by the differing risk–benefit profile of GLP-1 RA treatment in patients with obesity depending on whether sarcopenia is present [60].

Use of advanced techniques, such as DEXA scans or bioelectrical impedance analysis (BIA), may be appropriate for high-risk patients, particularly those with pre-existing sarcopenia or those undergoing aggressive weight loss interventions. DEXA scans provide precise measurements of body composition, including lean mass and fat mass, allowing clinicians to track changes over time and make informed decisions about treatment adjustments. BIA, while less precise than DEXA, is a more accessible and cost-effective tool for evaluating body composition and monitoring changes in muscle mass [18].

The implementation of a comprehensive geriatric assessment (CGA) is also recommended for older patients undergoing weight loss interventions. The CGA involves a multidisciplinary evaluation that includes medical, nutritional, functional, and psychological assessments, which help to identify risk factors for frailty and guide individualized treatment plans. This holistic approach ensures that any potential issues, such as malnutrition, depression, or cognitive decline, are addressed early and appropriately [61]. It also optimizes interdisciplinary interventions, which may involve dietitians, physiotherapists, and geriatricians working collaboratively to ensure the patient’s nutritional, physical, and emotional needs are met.

Monitoring should be continuous, with therapy adjustments made based on the patient’s functional and nutritional trajectories. Regular follow-ups are crucial to assess the effectiveness of interventions and make necessary modifications. This approach ensures that weight loss interventions, particularly those involving GLP-1 RAs, do not result in unintended consequences, such as excessive muscle loss or functional decline, and that the overall health and quality of life of older women are preserved [18,62]. By combining resistance exercise, protein supplementation, and a comprehensive assessment and monitoring strategy, clinicians can help mitigate the risks of muscle loss during weight loss, thereby optimizing health outcomes for older women.

### 6.2. Resistance Exercise

Resistance exercise, typically performed 2–3 times per week (150 min/week), is considered the most effective intervention to preserve and enhance muscle mass during weight loss, particularly in older adults. This form of exercise induces mechanical loading on muscles, which stimulates muscle protein synthesis and counteracts the catabolic effects of caloric restriction, a common concern in weight loss strategies [63]. Resistance training, when performed correctly, promotes muscle hypertrophy and strength by creating a stimulus for the muscle fibers to adapt and grow. Additionally, this form of exercise is crucial in preventing the muscle atrophy that often accompanies weight loss, especially when combined with GLP-1 RAs, which facilitate fat reduction but may also result in lean mass loss [64].

Programs that incorporate progressive overload—gradually increasing the resistance or intensity of exercises—are particularly effective in promoting long-term muscle preservation and functional improvements. Functional movements, such as squats, lunges, and liftings, not only preserve muscle mass but also improve overall physical performance, thereby enhancing the individual’s capacity to perform daily activities. Evidence suggests that combining resistance exercise with GLP-1 RAs not only helps preserve lean mass but also leads to improvements in physical performance metrics, such as mobility, balance and strength [65]. Furthermore, resistance exercise improves insulin sensitivity, which is essential for managing obesity, diabetes, and metabolic syndrome [66]. Additionally, resistance training enhances mitochondrial function by increasing the number and efficiency of mitochondria within muscle cells, which is particularly important in older adults, as mitochondrial dysfunction contributes to muscle loss and metabolic decline [67]. Regular exercise, therefore, supports long-term weight maintenance by improving metabolic flexibility, which is the body’s ability to switch between burning fat and carbohydrates depending on availability.

### 6.3. Protein Supplementation

Adequate protein intake is critical in maintaining muscle mass, particularly in older adults who are undergoing weight loss interventions. The recommended protein intake for older women is generally 1.2–1.5 g of protein per kilogram of body weight per day. This higher protein requirement is due to age-related anabolic resistance, where muscle protein synthesis is impaired despite adequate dietary intake [68,69,70]. Leucine, a branched-chain amino acid, plays a pivotal role in stimulating muscle protein synthesis via the mTOR (mechanistic target of rapamycin) pathway. Leucine-enriched protein sources and essential amino acid supplements have been shown to enhance this anabolic response, further supporting muscle preservation during weight loss [71].

When combining GLP-1 RAs with protein supplementation, particularly in older women, optimal body composition outcomes can be achieved. This combined approach may mitigate the potential loss of muscle mass associated with GLP-1 RA therapy. Moreover, distributing protein intake evenly across meals has been found to be particularly effective, as it maximizes muscle protein synthesis throughout the day [72]. Consuming protein shortly after exercise has also been shown to enhance the anabolic response, facilitating muscle repair and growth. In clinical practice, it is essential to assess an individual’s dietary protein intake, especially during weight loss interventions, as deficiencies may exacerbate muscle loss and impair recovery [73]. Clinical guidelines recommend monitoring protein intake and providing supplementation or dietary adjustments as needed to ensure adequate protein consumption, particularly in older adults who may have decreased appetite or difficulty in obtaining sufficient protein from food alone [74].

All the abovementioned interventions are summarized in Table 2.

## 7. Conclusions and Future Perspectives

Data from randomized controlled trials (RCTs) and early real-world studies have demonstrated that GLP-1 receptor agonists (GLP-1 RAs) maintain their efficacy even when used in women over 65 years of age. However, several concerns remain regarding their use in this patient population, primarily related to the potential sarcopenic effects of these agents and their consequent impact on patients’ quality of life. While the reduction in lean body mass and functional capacity in younger individuals may appear marginal compared with the metabolic benefits associated with weight loss [75], in older adults this reduction may be crucial, potentially leading to loss of independence and the development of clinical frailty, with all the associated adverse consequences [76].

Optimal management therefore requires a personalized, geriatric-oriented approach in which pharmacological treatment is embedded within structured pre-conditioning strategies, including resistance-based physical activity and adequate protein supplementation, to preserve muscle mass, strength, and functional independence. Comprehensive geriatric assessment and longitudinal functional monitoring are essential to guide treatment intensity and duration.

Finally, ad hoc studies in patients with sarcopenic obesity are warranted to determine whether integrated protocols including GLP-1 RA therapy may prove superior, not only in terms of weight loss but also in overall functional and metabolic outcomes, compared with lifestyle interventions alone.

## Figures and Tables

**Table 1:** Key randomized controlled trials of GLP-1–based therapies for obesity: efficacy and evidence in older/female subgroups.

Trial	Drug	Design/Duration	Population (Overall)	Main Efficacy Results (Overall Population)	Older/Female Subgroup Data (If Available)	Main Conclusions
STEP 1	Semaglutide 2.4 mg	Phase 3 RCT; ~68 weeks	Adults with obesity or overweight without diabetes	Mean weight loss ~10–15%; significant improvement in cardiometabolic risk factors	Subgroup analyses including women and adults ≥ 65 years showed weight loss broadly comparable to overall population, with slight attenuation at advanced age	Semaglutide induces clinically meaningful weight loss with consistent efficacy across age and sex subgroups, though geriatric-specific outcomes are limited
STEP 2–4	Semaglutide 2.4 mg	Phase 3 RCTs; ~68 weeks	Adults with obesity or overweight, with or without metabolic comorbidities	Consistent double-digit weight reduction and metabolic improvement across trials	Age- and sex-stratified analyses generally consistent; detailed data in women ≥ 65 years variably reported	The STEP program confirms robust and reproducible efficacy; extrapolation to older women relies on subgroup consistency
STEP 5	Semaglutide 2.4 mg	Phase 3 RCT; ~104 weeks	Adults with obesity or overweight	Sustained weight loss over 2 years with durable metabolic benefits	Older adults and women maintained long-term benefit; modest attenuation reported in very old participants	Provides long-term evidence supporting chronic use, highlighting the need to preserve muscle mass in older women
SURMOUNT-1	Tirzepatide	Phase 3 RCT; ~72 weeks	Adults with obesity or overweight without diabetes	Mean weight loss ~15–21%; marked improvement in metabolic risk profile	Older adults included; age-stratified analyses showed efficacy comparable to overall population; female-specific geriatric data limited	Tirzepatide achieves greater weight loss than GLP-1 RAs alone, with potential relevance for older women
SURMOUNT-2	Tirzepatide	Phase 3 RCT; ~72 weeks	Adults with obesity or overweight and type 2 diabetes	Mean weight loss ~15%; significant HbA1c and cardiometabolic improvements	Subgroup analyses suggest preserved efficacy in older adults; specific data in women ≥ 65 years limited	Confirms strong efficacy in obesity with diabetes; individualized assessment is required in older women

RCT: randomized controlled trial.

**Table 2:** Multidomain and multidisciplinary approach to sarcopenia in women during weight loss.

✓ Consider GLP-1 RAs in Women > 65 Years in Presence of Obesity with Significant Cardiometabolic Burden
✓ Initiate concurrent resistance training and ensure adequate protein intake
✓ Monitor for signs of sarcopenia and functional decline
✓ Tailor dose and duration based on functional trajectory and patient goals
✓ Engage a multidisciplinary team (geriatrician, dietitian, physiotherapist, etc.)
✓ Screen for frailty using validated tools (e.g., Fried criteria, Clinical Frailty Scale)
✓ Adjust expectations and treatment intensity based on patient preferences, life expectancy, and comorbidities

## Data Availability

No new data were created.
